# The Sigma Enigma: A Narrative Review of Sigma Receptors

**DOI:** 10.7759/cureus.35756

**Published:** 2023-03-04

**Authors:** Joseph Pergolizzi, Giustino Varrassi, Mark Coleman, Frank Breve, Dana K Christo, Paul J Christo, Charbel Moussa

**Affiliations:** 1 Cardiology, Native Cardio Inc., Naples, USA; 2 Research, Paolo Procacci Foundation, Roma, ITA; 3 Pain Management, National Spine and Pain Centers, Frederick, USA; 4 Department of Pharmacy, Temple University, Philadelphia, USA; 5 Internal Medicine, Johns Hopkins University School of Medicine, Baltimore, USA; 6 Anesthesiology and Critical Care, Johns Hopkins University School of Medicine, Baltimore, USA; 7 Neurology, Georgetown University Medical Center, Washington, DC, USA

**Keywords:** alzheimers, psychiatric disorders, cancer, protein folding, cholesterol homeostasis, mitochondrial bioenergetics, calcium homeostasis, protein chaperones, receptors, sigma

## Abstract

The sigma-1 and sigma-2 receptors were first discovered in the 1960s and were thought to be a form of opioid receptors initially. Over time, more was gradually learned about these receptors, which are actually protein chaperones, and many of their unique or unusual properties can contribute to a range of important new therapeutic applications. These sigma receptors translocate in the body and regulate calcium homeostasis and mitochondrial bioenergetics and they also have neuroprotective effects. The ligands to which these sigma receptors respond are several and dissimilar, including neurosteroids, neuroleptics, and cocaine. There is controversy as to their endogenous ligands. Sigma receptors are also involved in the complex processes of cholesterol homeostasis and protein folding. While previous work on this topic has been limited, research has been conducted in multiple disease states, such as addiction, aging. Alzheimer’s disease, cancer, psychiatric disorders, pain and neuropathic pain, Parkinson's disease, and others. There is currently increasing interest in sigma-1 and sigma-2 receptors as they provide potential therapeutic targets for many disease indications.

## Introduction and background

In describing sigma receptors, the challenge is not that so little is known about these enigmatic proteins, but rather that much of what we thought we knew has turned out to be wrong. First discovered in the 1960s along with the mu-, kappa-, and delta-opioid receptors [1], the two types of the then-named “sigma opioid receptors” turned out to not only be unrelated to those other opioid receptors but also not be opioid receptors at all [2]. The sigma-opioid-receptor was renamed the sigma receptor around 1995 when it was first cloned and shown to be markedly dissimilar to the canonical opioid receptors and did not respond to classic opioid antagonists such as naloxone or nalmefene [3,4]. Another distinction was that while the mu-, kappa-, and delta receptors had an affinity for the (-) enantiomers of benzomorphans, the sigma receptors possess an affinity for the (+) as well as the (-) enantiomers [4,5]. Encoded by the SIGMAR1 gene in humans, the sigma-1 receptor is not a receptor in the conventional sense; rather, it is a chaperone protein that resides in the endoplasmic reticulum [6]. But even among chaperone proteins, sigma-1 is an outlier. It is the only known chaperone protein that is regulated by the agonist/antagonist activity of endogenous or synthetic compounds, making it act like a receptor [7]. There is some controversy about the endogenous ligand(s) for sigma-1 receptors, and there is no consensus that one has been unequivocally identified [8]. Neurosteroids, such as pregnenolone sulfate and progesterone, are likely ligands, but there may be others as well [9].

**Figure 1:**
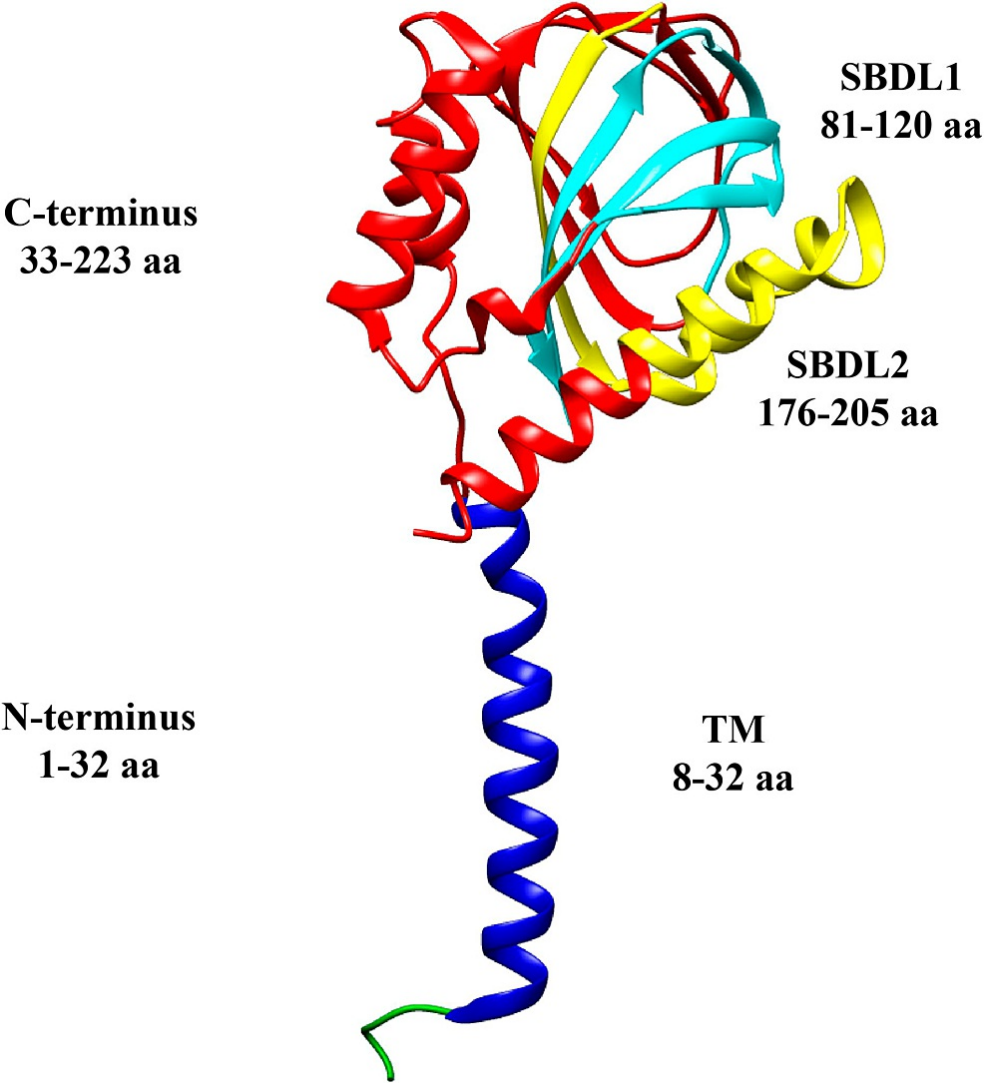
The topology of a sigma-1 receptor The N-terminus is green and blue, the transmembrane segment is shown in blue, and the C-terminus is red. The steroid binding domain-like (SBDL) regions are shown in aqua for 1 and yellow for 2, and both are located on the C-terminus The original art is licensed under Creative Commons and comes from [11]

The sigma-1 receptor retained the name “receptor,” even though its receptor activities are so unconventional that the term receptor may be a misnomer. The architecture too was different from expected: the sigma-1 receptor possesses a fold with a single transmembrane helix in each protomer, giving it a trimeric arrangement. Each protomer is capable of binding to a single molecule of the ligand at the center of its carboxy-terminal domain (Figure 1) [10].

Sigma receptors are proteins that are unlike any other protein in humans. In fact, sigma-1 receptors bear no resemblance to any other proteins found in mammals. The closest homolog to sigma-1 receptors is yeast (C8-C7 sterol isomerase, ERG2p). This itself is perplexing, as the sigma-1 receptors have no isomerase activity [3]. 

Sigma-1 receptors are present in the mitochondrion-associated endoplasmic reticulum membrane (MAM) within a cell [7]. When a ligand encounters a sigma-1 receptor, the sigma-1 receptor translocates from the MAM into the plasma membrane where it can then interact with the various ion channels and G-protein coupled receptors [7]. Mechanistically speaking, the sigma-1 receptor acts more like a signaling modulator between organelles than a classic receptor [6]. Functionally speaking, sigma-1 receptors are versatile; they are involved in lipid metabolism, lipid transport, gene transcription, modulation of voltage-gated ion channels (sodium, potassium, and calcium), regulation of non-voltage-gated ion channels, intracellular calcium homeostasis, control of oxidative stress, and the regulation of electrical activity in the cell and possibly other functions [6,12]. This list of functions may even be not complete.

However, much less is known about the counterpart sigma-2 receptor, which may not even be related to the sigma-1 receptor. The relationship between sigma receptors remains unknown because the sigma-2 receptor has never been cloned and its structure is unknown. 

The objective of this article is to present a short narrative review of the current understanding of sigma receptors and how our evolving elucidation of these proteins may offer important therapeutic drug targets in the future. 

Methods 

The authors searched PubMed and Google Scholar using the keywords “sigma receptor,” “sigma-1 receptor,” and “sigma-2 receptor.” The bibliographies of some of the articles were also searched. The goal was to create a succinct narrative review of a complex and rapidly evolving topic.

## Review

The sigma-1 receptor exists in monomer, dimer, tetramer, hexamer/octamer, and high oligomeric forms, but it is not known if these conformational differences affect functionality. Only one portion of the sigma-1 receptor binds to the ligand. The chaperone function may involve co-chaperone 78-kDa, a glucose-regulated protein [13]. Sigma-1 receptors respond to ligands as agonists or antagonists [8]. Once described as “orphan receptors,” sigma-1 receptors have long remained elusive and the few that have been proposed are controversial [7,14]. Suggested as endogenous ligands of sigma-1 receptors are sphingosine, N, N-dimethyl sphingosine (DMS), dehydroepiandrosterone (DHEA), N-dimethyltryptamine (DMT), and progesterone [13]. Brailoiou et al. propose that choline may also be a native ligand [15]. While endogenous ligands are elusive, the sigma-1 receptor binds promiscuously to a variety of dissimilar exogenous ligands, such as dextromethorphan, haloperidol, fluoxetine, quetiapine, clemastine, and chloroquine [2]. While these ligand molecules tend to share an elongated shape, positive charge, and hydrophobia in common, there are many unrelated groups: neurosteroids, neuroleptics, and psychostimulants such as cocaine and methamphetamine among them [16].

To understand these little-understood receptors, this review will briefly describe various relevant portions of the cell and cellular processes. They are presented in what we consider a logical order for understanding these processes:

The endoplasmic reticulum

Both sigma receptors play a role in lipid synthesis that has not yet been elucidated and sigma-1 receptors appear to be densely localized in cholesterol-rich areas of the body [17]. Lipid biogenesis takes place within the cell near the endoplasmic reticulum and Golgi apparatus. As lipids are metabolized, they move throughout the cell, either finding their place within the cell or being sent out into the body via secretory vesicles [18]. 

The endoplasmic reticulum likewise acts as a storehouse for intracellular Ca2+ and contains multiple calcium channels allowing for the ingress of the calcium ions into the cytosol when calcium levels decrease. Calcium ions serve as signaling molecules as well, affecting any number of functions, including neuronal processes [19].

Bioenergetics within the body involves the mitochondrion working together with the endoplasmic reticulum at the MAM interface. The endoplasmic reticulum contributes calcium to the mitochondrion via inositol 1,4,5-triphosphate (IP3) receptors. A sigma-1 receptor creates a complex with the immunoglobulin heavy-chain-binding protein (BiP) chaperone located in the MAM; if calcium levels in the endoplasmic reticulum decrease, the sigma-1 receptor dissociates from the BiP. Sigma-1 receptors translocate readily when the endoplasmic reticulum is being affected by prolonged stressors. In such instances, the translocation of a large number of sigma-1 receptors into the endoplasmic reticulum can effectively counter that stress. Conversely, when an insufficient number of sigma-1 receptors respond, apoptosis can occur [7]. 

Chaperones 

A chaperone protein guides other proteins during synthesis, folding, and/or function [20]. Protein folding, which occurs in most living beings including humans, starts when proteins are synthesized in the endoplasmic reticulum. Nascent proteins enter the endoplasmic reticulum as linear peptide chains via the translocons, an aqueous pore that allows proteins to move from the cytoplasm into the lumen of the endoplasmic reticulum. A translocon is not a fixed “porthole,” but rather a dynamic and changing structure with highly nuanced actions [21]. Once the peptide chains enter the endoplasmic reticulum, they are folded into the proper three-dimensional configuration and can be transported out of the endoplasmic reticulum to their final destination. Chaperone proteins in the endoplasmic reticulum, including but not limited to sigma-1 receptors, are tasked with the proper folding of the nascent proteins [20]. The endoplasmic reticulum also maintains what might be considered a quality control function, which searches out terminally misfolded polypeptide chains, so that they may then be removed by autophagy or endoplasmic-reticulum-associated degradation (ERAD) [22]. Improper folding may be the result of biological insults, insufficient glucose, DNA damage, viral attack, free radicals, intoxication, and other factors [20]. Some chaperones can repair misfolded proteins. A series of misfolding can trigger the “unfolded protein response” (UPR) to restore appropriate folding [20]. An accumulation of misfolded proteins causes stress in the endoplasmic reticulum, which has been implicated in the pathogenesis of cancer, neurodegenerative diseases, diabetes, and other conditions [23]. Indeed, Parkinson’s disease may be described as a protein-folding disorder [24]. UPR is a signal transduction pathway that reduces the burden of unfolded proteins and promotes cellular viability [23]. Recent research suggests that this adaptive UPR response has functions apart from protein folding associated with lipid metabolism and bioenergetics [23]. 

Mitochondria**

Mitochondria, described as “chemical energy powerplants,” are organelles that manage biogenesis and produce cellular energy [25]. Mitochondria possess their own DNA (mtDNA), have multiple membrane compartments, and can migrate or undergo mitophagy to maintain metabolic homeostasis [26]. Mitochondria have been implicated in apoptosis but the mechanisms are not clear and remain controversial [25]. The sigma-1 receptor resides in eukaryotic mitochondrial-associated endoplasmic reticula and plasma membranes [13]. The interactions between the endoplasmic reticulum and the mitochondrion, two distinct organelles in the cell, have only recently been elucidated. The endoplasmic reticulum connects to the mitochondrion by way of MAM, a membrane that is part of the endoplasmic reticulum. MAM facilitates communication between the endoplasmic reticulum and the mitochondrion by way of the large CA2+ ions. The endoplasmic reticulum transports CA2+ ions through MAM directly into the mitochondrion. This CA2+ is used in protein biosynthesis and to maintain chaperone activities in the ER. This form of CA2+ transport via MAM is far more efficient than having the mitochondria import CA2+ from the cytosol [27]. Thus, MAM may be viewed as a bridge between the endoplasmic reticulum and the mitochondrion to help regulate calcium homeostasis, energy exchange, lipid synthesis, lipid transport, and to manage protein folding [27].

The mitochondria are revealing themselves to be far more than cellular powerplants; they also can trigger a variety of complex signaling cascades [28]. Mitochondrial dysfunction can launch a variety of adaptive responses known as the mitochondrial death cascade. During the death cascade, the mitochondria trigger the release of reactive oxygen species (ROS), reactive nitrogen species (RNS), caspases, and pro-apoptotic actions. Sigma-1 receptor activation can confer neuroprotection in that it may preserve the anti-apoptotic genes (such as bcl-2) and regulate intracellular calcium levels [29]. Mitochondrial dysfunction is emerging as a pathological process of interest in our growing understanding of the mechanisms underlying neurodegeneration [30]. 

Autophagy**

Autophagy is a normal and health-promoting catabolic process by which cells can get rid of debris and can eliminate compromised portions of the cell or misfolded proteins. The role of sigma-1 receptors in autophagy is not clear but may be of great importance [31]. The macrophages of the central nervous system are microglia which preserve homeostasis in the brain. These versatile microglia can change into response phenotypes M1 or M2 based on the situation [32]. M1 microglia are pro-inflammatory and promote degeneration, and, conversely, M2 are anti-inflammatory and promote regeneration [33]. Neuroinflammation can exert either a damaging or protective effect on the brain in different circumstances [33]. Microglia are of interest in this context because they contain both sigma-1 and sigma-2 receptors that may be able to mitigate M1 effects [34].

Ligands

The sigma-1 receptor has been described as a ligand-activated chaperone protein rather than a typical receptor [7]. Our current understanding of sigma-1 receptors maintains that, in the resting state, they reside in the MAM of the endoplasmic reticulum [27] and form a complex with the binding immunoglobulin protein (BiP), another chaperone likewise involved in protein folding and protein quality control [27]. Decreased calcium levels or small-molecule agonists in the endoplasmic reticulum cause the sigma-1 receptors to detach from the BiP and interact with client proteins in the endoplasmic reticulum or other organelles [27]. 

The sigma-1 receptor is capable of binding to many different proteins, including voltage-gated sodium channels, voltage-gated calcium channels, N-methyl-D aspartate receptors (NMDAR) at the GluN1 and GluN2 subunits, ghrelin, dopamine transporters, and others [8]. The electrostatic interaction between Glu172 and nitrogen present in the ligand is the primary way in which sigma-1 receptors bind with their ligands, which explains why they are able to bind effectively with many structurally dissimilar ligands. The actual binding site is a small area on the molecule. Most of these ligands have a crystal structure, and, after binding, the remaining portion of the ligand fits into the binding pocket of the receptor, which is lined with hydrophobic residues [5]. 

Agonists and antagonists**

Both sigma-1 and sigma-2 receptors appear to react to certain exogenous substances as an agonist or an antagonist, making them similar to receptors although they are protein chaperones. See Table 1. 

**Table 1:** Agents that may act as an agonist or antagonist at sigma-1 or -2 receptors* *[27,35,36] Note that some of these substances are drugs in development and are not commercially released

	Sigma-1 receptors		Sigma-2 receptors	
	Agonist	Antagonist	Agonist	Antagonist
ADV502		X		
BD-1063	X			
CT01344A		X		
CT1812				X
Cutamesine (SA4503)	X			
Dextromethorphan	X			
DHEA sulfate	X			
Donepezil	X			
Fluvoxamine	X			
Haloperidol	X			
MR309	X			
PB221			X	
Rimcazole	X			
Roluperidone (MIN-101)				X
Siramesine (Lu28-179)		X	X	

Calcium signaling**

The most prominent function of sigma-1 receptors is modulating voltage-regulated and ligand-gated ion channels for CA2+, K+, NA+, small-conductance calcium-activated potassium channels (SK), and for receptors of NMDA and triphosphate inositol (IP3) receptors [37]. Of particular interest are the enigmatic IP3 receptors, which appear to be clients of the sigma-1 receptors [20]. The sigma-1 receptor locates itself near the IP3 receptor, typically at or near MAM, the junction of the endoplasmic reticulum and mitochondrion. When stimulated, these receptors can open and generate high concentrations of CA2+ to be delivered to the mitochondria; however, IP3 receptors degrade quickly [20]. The sigma-1 and IP3 receptors coimmunoprecipitate, which suggests interacting with each other directly [7]. The IP3 receptor can also colocalize and coimmunoprecipitate with other receptors that act as chaperon proteins for the endoplasmic reticulum, such as GRP78 or BiP [7]. One of the main roles of the sigma-1 receptor is to stabilize the conformation of the IP3 receptor, which makes it more robust and resistant to degradation. When the IP3 receptor opens, the calcium levels in that immediate area of the endoplasmic reticulum decrease. The sigma-1 receptor dissociates itself from the BiP and translocates to the IP3 receptor to serve as an IP3 receptor chaperone. It is thought that this activity assures that calcium signaling to and from the endoplasmic reticulum and the mitochondrion are traveling appropriately through the MAM [7]. 

In the case that a pathological condition might deplete the calcium levels at the endoplasmic reticulum lumen for a prolonged time, the sigma-1 receptor will leave the MAM and translocate through the entire endoplasmic reticular network to increase calcium levels, even to the point that the calcium levels of the endoplasmic reticulum are nearly depleted. This activity can increase cell survival by reducing the chances of low levels of calcium triggering widespread apoptosis [7]. Thus, it is fair to describe the sigma-1 receptor as a chaperone that promotes proper calcium signaling and protects the cell from apoptosis when prolonged calcium depletion threatens the cell’s survival [7]. The sigma-2 receptor is thought to chaperone the NPCI gene [38]. 

Cholesterol 

The disruption of lipids in the body appears to be related to several neurodegenerative diseases [39]. Cholesterol and other lipids are not uniformly distributed throughout the plasma membrane, and the theory is that the body forms “lipid rafts” or microdomains form of gangliosides, sphingomyelin, and cholesterol [40,41]. The MAM may be considered an example [42]. Because not all lipids and forms of cholesterol interact well with each other, it has been considered that these so-called “rafts” may be more aptly described as layers with forces of attraction and repulsion involved [43]. 

While about half of the brain's weight is made up of cerebral lipids, the blood-brain barrier prevents cholesterol uptake from the body into the brain [44]. Cholesterol homeostasis plays a major role in health and these balances are maintained independently in the peripheral nervous system and in the brain. Thus, the brain manufactures its own cholesterol, by way of the lipid carrier, apolipoprotein E (ApoE) [45,46]. ApoE is a glycoprotein and is produced in the peripheral tissue as well [47]. This relatively inefficient process recruits external astrocytes to the task. ApoE plays a role in cardiovascular health and the risk for Alzheimer’s disease, as well as hyperlipidemia [47,48]. Crosstalk between the peripheral and central nervous systems with respect to cholesterol regulation remains an important area for future study, as cholesterol derangement is associated with metabolic as well as neurodegenerative disorders [49]. 

The unfolded protein response

As the largest organelle of the eukaryotic cell, the endoplasmic reticulum has many important functions, including lipid and steroid synthesis, carbohydrate metabolism, calcium storage, and protein synthesis and folding [50]. The endoplasmic reticulum is capable of changing shape to accommodate these functions [50]. Protein synthesis requires ribosomes to localize around the cytosolic face of the endoplasmic reticulum which allows the mRNA-ribosome complex to dock into the endoplasmic reticulum membrane. With the aid of specialized enzymes and chaperone proteins, these proteins must first be folded [50]. Misfolded or otherwise aberrant proteins may avoid secretion to their ultimate destinations and remain in the endoplasmic reticulum. Over time, deficient proteins can accumulate in the endoplasmic reticulum, which can launch the unfolded protein response (UPR), aimed at restoring protein homeostasis. The UPR is a signal transduction pathway that can trigger the apoptosis of damaged cells. Prolonged disruption of proteostasis has been linked to neurodegenerative disorders and the UPR has been identified as an important therapeutic target [51]. Since the UPR is one of several signaling pathways involved in lipid regulation, lipid derangement can trigger UPR without misfolded proteins present [52]. 

Sigma-2 receptors**

Compared to sigma-1 receptors, much less is known about the sigma-2 receptors, and, in fact, it is not clear if they are even related to sigma-1 receptors, at least genetically [8]. The binding site of the sigma-2 receptor has been identified as the endoplasmic-reticulum-resident transmembrane protein (TMEM) 97, sometimes called the meningioma-associated protein (MAC) 3. The sigma-2 receptors are related to TMEM97 and the emopamil binding protein (EBP), which is the mammalian form of C8-C7 sterol isomerase (the most closely related isomerase to sigma-1 receptors) [8,53]. Sigma-2 receptors are involved in cholesterol synthesis and homeostasis [45]. 

The sigma-2 receptor has never been cloned [54] and its structure is unknown [55]. Sigma-2 receptors have a molecular weight in the range of 18 to 21.5 kD in contrast to the sigma-1 receptors, the molecular weight of which is 25 kD.55. Sigma-2 receptors are overexpressed in tumors and their prominent expression may be considered a biomarker for certain malignancies [56]. 

Sigma receptors as therapeutic targets 

It goes beyond the scope of this article to describe in detail all of the pathologies and conditions in which sigma receptors have been implicated as playing a role. Some highlights of conditions being investigated with respect to sigma receptors, in alphabetical order, are as follows:

Addictions 

Substance use disorders associated with stimulants such as cocaine and methamphetamine may trigger persistent changes in cellular electrical activity known as cellular intrinsic plasticity [57,58]. Substance use disorders further cause neuroplastic changes to the brain associated with reinforcement and reward circuits. Sigma-1 receptors play a role in the cellular electrical plasticity that underlies stimulant addiction [37]. Methamphetamine and cocaine both bind at the sigma-1 receptors with moderate affinity and the drug’s effects may be blunted or blocked entirely with a sigma-1 antagonist [59,60]. Note that the endogenous hormone progesterone may be a sigma-1 receptor antagonist that can effectively reduce cocaine cravings [61,62]. The connection between sigma-1 receptors and alcohol use disorder has not been elucidated [62]. However, sigma receptors may be a promising therapeutic target as they help modulate the rewarding and reinforcing effects of substances [63,64].

Aging 

The “graying” of developed nations has raised great interest in anti-aging products and treatments, but aging is such a complex and multifactorial process that depends on the interplay of multiple factors that it has been difficult to clearly define “the” aging mechanisms among the cascade of aging processes. Research into sigma receptors suggests that proteostasis, the proper folding, chaperoning, and maintenance of proteins in the cell may be key drivers [65].

Alzheimer’s Disease 

The current model of Alzheimer’s disease maintains that amyloid-β deposits and neuro-fibrillatory tangles of hyperphosphorylated tau proteins accumulate in the brain. In a postmortem study, Alzheimer’s disease patients had fewer sigma-1 receptors in the CA1 stratum pyramidale region of the hippocampus when compared to those without Alzheimer’s disease [66]. It is believed that the loss of sigma-1 receptors in the brain occurs early in the course of Alzheimer’s disease, mainly affecting the frontal, temporal, and occipital lobes [67]. There is evidence that sigma-1 receptor agonists can exert a neuroprotective effect on the brain, which, in turn, may reduce cognitive deficits [6].

Sigma receptors may be therapeutic targets for Alzheimer’s disease because of their role in cholesterol homeostasis [45]. Since the blood-brain barrier prevents cholesterol uptake into the brain, external astrocytes are recruited to help the brain produce its own cholesterol [46]. While cholesterol biosynthesis declines in patients with Alzheimer’s disease [68], cholesterol levels within the cells paradoxically increase [69]. Thus, high cholesterol levels have been suggested as a biomarker for Alzheimer’s disease and the risk for Alzheimer’s disease decreases in those taking cholesterol-lowering statin drugs [70-72]. Moreover, lipid composition in the body is altered with aging, and changes in fatty acids and cerebral lipid peroxidation have been noticed in the brains of individuals with early-stage Alzheimer’s disease [39]. So closely is lipid metabolism related to Alzheimer’s disease that it has given rise to the study of lipidomics, the study of lipid regulation, dietary lipid intake, and environmental factors related to lipids and their effect on Alzheimer’s disease [39].

In humans, cerebral lipids increase over the first 20 years of life, plateau until about age 50, and then gradually decrease [73]. The most abundant polyunsaturated fatty acid in the human brain is docosahexaenoic acid (DHA). DHA decreases with age. Low levels of DHA are a hallmark of Alzheimer’s disease and these lower DHA levels are associated with hippocampal atrophy and correlated with dementia [74]. 

Both sigma receptors are involved with lipid homeostasis, and inhibition of sigma-2 receptors can bind neurons and may reduce cognitive dysfunction associated with Alzheimer’s disease [75]. Sigma-2 antagonists in particular appear to halt the amyloid cascade associated with Alzheimer’s disease in which beta-amyloids accumulate in the brain [76,77].

Amnesia 

Sigma-1 receptor agonists are able to potentiate NMDA-driven neuronal firing into the CA3 hippocampal region of the brain [78]. These agonists can increase extracellular acetylcholine levels in the hippocampus or cortex of rat brains during intracerebral microdialysis [37]. 

Amyloid Lateral Sclerosis 

Amyloid lateral sclerosis (ALS) is a rare, fatal, progressive neurodegenerative disorder for which there is increasing evidence of mitochondrial dysfunction [79]. Both mitochondrial dysfunction and the degeneration of motor neurons have been associated with oxidative stress that could lead to pathological mitochondrial dysfunction. Oxidative stress can cause abnormal aggregation of proteins as well as mutations in mitochondrial DNA. The role of sigma-1 receptors in ALS may be its ability to modulate the calcium balance and reduce oxidative stress [79]. 

ALS can be heritable or sporadic [80]. Genetic polymorphisms have been discovered in the gene that encodes the sigma-1 receptor, making a sigma-1 receptor agonist a plausible treatment target [81]. A homozygosity mapping study that sequenced the sigma-1 receptor gene revealed a mutation in the transmembrane domain that could be associated with juvenile ALS [82]. 

Cancer 

A well-known characteristic of cancer is an increase in oncogenes and a concomitant decrease in tumor suppressors [83]. Cancer cells may also trigger an increase in cholesterol uptake and produce oxysterols, which can induce apoptosis and tumor cell death [84]. The production of oxysterols may result in the overproduction of ROS and/or an increase in CA2+ levels in the body. Sigma-2 receptors are densely expressed in proliferating cancer cells, but less so in quiescent cancer cells, making them a potentially useful biomarker [57]. Moreover, sigma-2 ligands may have cytotoxic properties and be able to inhibit cancer cell growth and even trigger tumor apoptosis [85]. 

The beneficial role of sigma-2 ligands in cancer treatment extends to their role in cholesterol homeostasis [45]. There is a putative link between cell signaling and lipid rafts [86]. A model has been proposed showing how lipid raft modeling by sigma-1 receptors occurs in breast cancer lines. The COOH terminus of the sigma-1 receptor has a cholesterol-recognition domain; cholesterol binds to this area. Inhibiting the sigma-1 receptor prevents cholesterol binding [87]. Active cancer cells building new membranes and signaling networks depend on the production of endogenous cholesterol and uptake of lipid particles to the point that accelerated cholesterol metabolism has become known as a hallmark of cancer. Cholesterol and other lipids contribute to malignancies [88]. 

Tumor cells have been observed to overexpress sigma-2 receptors and sigma-2 agonists may induce cytotoxicity in cancer cells via the disruption of both lysosomal function and ROS production [89]. The role of calcium regulation by the sigma-2 receptor may also play a role in its anticancer properties [89]. This field of research holds great promise, and other studies would be useful.

Cardiovascular Diseases 

Dysfunctions in the MAM have been related to cardiovascular disease and the appropriate ongoing bidirectional communication between the mitochondria and the endoplasmic reticulum serves to regulate CA2+ homeostasis and lipid biosynthesis, essential to heart health [90]. Following oxidative stress to the cardiomyocytes, sigma-1 receptors can help regulate the “clean-up” process of autophagy [91].

Chemotherapy-Induced Peripheral Neuropathy 

Sigma-1 receptor inhibition has been shown in animal studies to block many forms of neuropathic pain [92]. Based on promising preclinical results, these inhibitors may be effective in treating paclitaxel-induced neuropathic pain [93] and oxaliplatin-induced neuropathic pain [94]. 

COVID Infection 

The recent interest in fluvoxamine, a sigma-1 receptor agonist, for the treatment of COVID infection and postviral syndrome long COVID was based on the observation that this agent can decrease SARS-CoV-2 replication, potentially reducing endoplasmic reticulum stress, and decreasing the related inflammatory response [95]. All coronaviruses replicate in a modified compartment in the endoplasmic reticulum, so it is logical that the resident chaperone proteins (sigma receptors) might be a potential therapeutic target [96]. 

Depression and Anxiety 

Although clinically distinct entities, depression and anxiety share certain pathophysiological mechanisms, of which the dysfunctions in the monoaminergic, GABAergic, and glutamatergic systems are most prominent [97]. Pharmacological treatments exist but are not always effective and may be associated with significant side effects. Sigma-1 receptor blockers may be important new treatment options as individual agents or adjuvants [97]. An interesting and related application of sigma-1 receptor inhibitors may be the relief of emotional suffering and cognitive deficits associated with chronic pain due to osteoarthritis [98]. Chronic pain, depression, and anxiety are closely linked [99], but it is clear the extent to which depression and anxiety might contribute overall to chronic pain syndromes. Furthermore, depression may be a prodromal harbinger of subsequent schizophrenia, so early intervention may help delay or prevent schizophrenia [100]. 

*Neuropathy* 

Following peripheral nerve injury, the affected primary sensory neurons exhibit a dense expression of sigma-1 receptors, which play a crucial part in modulating the nociceptive functions of the peripheral sensory system. However, the increased number of sigma-1 receptors induced by nerve injury may drive neuropathic pain instead [101]. Obesity-induced peripheral neuropathy is not well studied, but murine studies suggest that the sigma-1 receptor may enhance NMDAR expression in the spine and, in that way, mediate the condition [102]. In a study of rats, sigma-1 receptor blockade prevented peripheral diabetic neuropathy [103]. Sigma-1 receptors may confer a neuroprotective effect against glaucoma, an optic neuropathy that shares certain pathologies in common with Alzheimer’s disease and Parkinson’s disease [104]. Sigma-1 receptor agonism in this case may enhance neuronal plasticity, and neurite growth, and serve as a new therapeutic target for glaucoma. This paradoxical situation where sigma-1 receptors serve to enhance both neurodegeneration and neuroprotection has been explained by Nguyen et al., who report that disease progression is exacerbated by dysfunction of sigma-1 receptors but neuroprotection is improved when sigma-1 receptor activity is enhanced [105].

Pain 

Historically, sigma receptors took their name from the dubious notion that they were another type of opioid receptor and thus involved in analgesia [106]. Nevertheless, the sigma receptors may be associated with opioid-mediated analgesia. The sigma-1 receptor antagonist, haloperidol, potentiates opioid analgesia while sigma-1 receptor agonists attenuate it [107]. It is thought that sigma-1 receptors influence opioid-induced analgesia at the supraspinal level [107]. 

Parkinson's Disease 

Alpha-synuclein oligomers can disrupt protein trafficking, autophagy, lysosomal function, protein clearance, and synaptic integrity and have been implicated in Parkinson's disease [108]. The spread of alpha-synuclein oligomers has been linked to disease progression. Sigma-2 receptor antagonists are effective blockers of alpha-synuclein oligomer-trafficking deficits. Thus, sigma-2 receptor antagonists have emerged as promising new drugs for Parkinson's disease and may reduce neurotoxicity [108].

Retinal Disease 

Many sigma-1 receptor functions appear to modulate biological functions related to degenerative disease of the retina, a major cause of blindness around the world [109]. The functions of interest are calcium regulation, control of oxidative stress, ion channel modulation, and molecular chaperone actions. The sigma-1 receptor plays a major role in regulating retinal cellular stress [109].

Schizophrenia 

Sigma-1 receptors regulate glutamatergic, dopaminergic, serotonergic, noradrenergic, and cholinergic systems, which may make them a potential drug target for schizophrenia treatments [110]. Schizophrenia is characterized by nonspecific prodromal syndromes, including depression and cognitive dysfunction, which in some individuals can transition into frank schizophrenia [100]. Sigma-1 receptor agonism with fluvoxamine has been proposed as a potential agent to reduce the risk of transition from prodromal syndrome to schizophrenia, although this has not been studied in a large clinical trial [111]. 

Stroke 

Stroke is known to cause damage to brain cells, from which recovery can be slow or incomplete. After ischemic stroke, the NMDAR plays a role in the excitotoxic neural damage that can follow; this can be regulated by sigma-1 inhibition [112]. Murine studies are currently ongoing. 

Discussion 

The sigma receptors 1 and 2 present myriad intriguing drug targets and science's incomplete elucidation of these receptors already has provided greater insight into lipid and protein synthesis, neuroprotective mechanisms, protein synthesis and folding, and many other processes. In describing the potentials of sigma receptors, there are very few areas of medicine that will not be affected.

While there is important research into sigma receptors, these efforts remain fairly limited. For instance, from 1992 to 2017, only 1,102 peer-reviewed articles on sigma receptors were published and only 247 authors have five or more publications [113]. (By contrast, from 1992 to 2017 there were 492,296 peer-reviewed articles published on diabetes; 28,423 on psoriasis; and 51,260 on ischemic stroke.) The majority of articles on sigma receptors come from the United States, Germany, and Japan. From a cluster analysis of these articles, it was found that the main focus of these articles was on addiction, psychiatric diseases, neurodegenerative disorders, and pain, although these do not represent the full therapeutic potential of these receptors [113]. 

Greater interest in the topic of sigma receptors, more research, more academic discussions, and more publications are urgently needed, as these receptors may offer a multitude of highly important drug targets for future development, plus a greater understanding of their function can improve our understanding of aging, neurodegeneration, cancer, and cholesterol synthesis, among others. There are scarcely any topics in modern medicine for which sigma receptors are not at least potentially of interest. Better collaboration among researchers and greater promotion of the topic of sigma receptors may be helpful. Healthcare professionals often know little about sigma receptors, but many still think of them as just another type of opioid receptor, a theory that has been debunked [114].

## Conclusions

Sigma-1 and sigma-2 receptors were once thought to be opioid receptors; today, they are understood to be protein chaperones that respond to ligand agonism or antagonism. These unique receptors are not well understood and as they become elucidated, a wide range of potential therapeutic applications from cancer to Alzheimer’s disease have emerged. Sigma-1 and -2 receptor agonists and antagonists are being developed, and the results have been promising, suggesting new therapeutic alternatives as well as elucidating previously enigmatic pathophysiological processes. Further research on the sigma receptors is urgently needed, as these agents may offer new breakthrough treatments for a range of diseases.
